# Driving Performance in Patients With Idiopathic Cervical Dystonia; A Driving Simulator Pilot Study

**DOI:** 10.3389/fneur.2020.00229

**Published:** 2020-04-03

**Authors:** J. van den Dool, B. Visser, R. B. Huitema, S. R. Caljouw, M. A. J. Tijssen

**Affiliations:** ^1^Faculty of Health, ACHIEVE Centre of Applied Research, Amsterdam University of Applied Sciences, Amsterdam, Netherlands; ^2^Department of Neurology, University Medical Centre Groningen, Groningen, Netherlands; ^3^Centre for Human Movement Sciences, University of Groningen, Groningen, Netherlands

**Keywords:** cervical dystonia, driving, driving performance, simulator, daily life tasks

## Abstract

**Objective:** To explore driving performance and driving safety in patients with cervical dystonia (CD) on a simulated lane tracking, intersections and highway ride and to compare it to healthy controls.

**Design:** This study was performed as an explorative between groups comparison.

**Participants:** Ten CD patients with idiopathic CD, 30 years or older, stable on botulinum toxin treatment for over a year, holding a valid driver's license and being an active driver were compared with 10 healthy controls, matched for age and gender.

**Main outcome measures:** Driving performance and safety, measured by various outcomes from the simulator, such as the standard deviation of the lateral position on the road, rule violations, percentage of line crossings, gap distance, and number of collisions. Fatigue and driving effort were measured with the Borg CR-10 scale and self-perceived fitness to drive was assessed with Fitness to Drive Screening.

**Results:** Except for a higher percentage of line crossings on the right side of the road by controls (median percentage 2.30, range 0.00–37.00 vs. 0.00, range 0.00–9.20, *p* = 0.043), no differences were found in driving performance and driving safety during the simulator rides. Fatigue levels were significantly higher in CD patients just before (*p* = 0.005) and after (*p* = 0.033) the lane tracking ride (patients median fatigue levels before 1.5 (range 0.00–6.00) and after 1.5 (range 0.00–7.00) vs. controls median fatigue levels before and after 0.00 (no range). No significant differences were found on self-perceived fitness to drive.

**Conclusion:** In patients with CD there were no indications that driving performance or driving safety were significant different from healthy controls in a simulator. Patients reported higher levels of fatigue both before and after driving compared to controls in accordance with the non-motor symptoms known in CD.

## Introduction

Cervical Dystonia (CD) is a neurological disorder characterized by involuntary muscle contractions causing abnormal positions and involuntary movements of the neck and head. With an estimated prevalence of 4.9 (CI95% 3.6–6.9) patients per 100,000 persons, it is the most common form of dystonia. (1) Besides motor symptoms, patients may also suffer from non-motor symptoms including pain, anxiety, depression, fatigue, and loss of self-confidence (2, 3). Both motor and non-motor symptoms can have a major impact on the level of disability causing limitations in daily life activities (4, 5). The preferred treatment is to inject the affected neck muscles with Botulinum Toxin (BoNT) injections which reduces involuntary movements and abnormal postures in 75–92% of the patients (6). Improvement of motor symptoms also decrease the level of disability but BoNT treatment is often not fully satisfactory and many patients still maintain difficulties while performing daily life tasks.

The ability to drive a vehicle is a very important part of daily life activities. It is important for social contacts, to get to work, and to access everyday needs such as goods and services. Driving a motor vehicle is a complex task requiring adequate interaction between visual, cognitive, and motor skills. It is known that a wide range of acute and chronic medical conditions may impair driving performance and safety (7). Since CD is characterized by involuntary movements and/or abnormal postures of the neck, patients may be impaired in visual scanning of the environment which may affect driving performance. In clinical practice some patients with CD indicate to use tricks or operational adaptations in the car, such as supporting their head while driving or turning their entire torso to the required direction before crossing an intersection. It is conceivable that patients also apply compensatory actions on a tactical level during driving, such as lowering speed or keeping appropriate distance. Furthermore, patients might compensate on a strategic level for example by adequate planning of a trip, avoiding rush hours or using automatic gear shift (8). The known fatigue problems in CD patients can also hamper their driving abilities (9). Currently there is no literature available on driving performance in CD.

A safe and standardized objective method to determine driving performance is with a driving simulator which enables to study real time driving performance and driving strategies in a controlled environment (7, 10). Driving performance is a concept that refers to the ability of a person to safely navigate a driving vehicle through traffic and can vary for different traffic situations. It can be measured by different outcomes such as vehicle control, speed adaptations to different traffic situations, distance control between the driver, and other traffic or gap acceptance time for crossing intersections or merging onto adjacent lanes. A subjective judgement of a participant's driving performance is the self-reporting questionnaire Fitness To Drive Screening (FTDS or previously called the Safe Driving Behavior Measure) (11). Based on the indications by patients in the clinical practice and the limitations in performing daily life activities, it is hypothesized that the operational driving skills are impaired and that CD patients have more difficulties in controlling the vehicle. To compensate for these impairments patients, when compared to healthy controls, might drive more carefully by lowering their speed, keeping more distance to a car in front or selecting a larger gap between approaching cars when crossing an intersection. It is also hypothesized that patients experience more fatigue and have to make more effort to drive safely compared to healthy controls. The main goal of this pilot study was therefore, to explore objective driving performance and the subjective driving evaluation (measured by the FTDS) in a small group of persons with CD and to compare it to healthy controls.

## Methods

### Study Design

The study was performed as an explorative between group comparison.

### Participants

Ten CD patients were recruited from the neurology department of the University Medical Center Groningen with the following inclusion criteria: idiopathic CD, 30 years or older, stable on BoNT treatment for over a year, holding a valid drivers' license and being an active driver. Participants were excluded if they had secondary or hereditary forms of dystonia, dystonia in other body parts than the neck, if they had had deep brain stimulation or selective nerve denervation for the treatment of their dystonia, suffered from other neck conditions or used medication that could affect driving performance. Healthy controls, matched for age and gender, holding a valid drivers' license, and active drivers were recruited among hospital staff and their relatives. Eligible participants had to understand the spoken and written Dutch language. Written informed consent was obtained from all participants. This study was approved by the local medical ethics committee (METC number 2013/507).

### Procedures

#### Data Collection

Measurements were performed 4–8 weeks after BoNT injections. With an average working period of 12 weeks and a peak effect between 2 and 6 weeks after injections, we expected an average effect of the BoNT injections (12).

The main parameters, driving performance and driving safety, were derived from a validated fixed based driving simulator located at the University Medical Center Groningen (ST Software Simulator Systems) (13). The simulator consisted of a projection screen stage and an open cabin mock-up standing within this stage and containing a force-feedback steering wheel, accelerator, clutch, brake pedals, and audio sound simulated driving sound. The projection screen stage held three video projectors and three large flat-screens of 4.5 m length, bent 60 degrees inwards, which presented the participants with a 180 degrees horizontal view. Front and side windows as well as a rear view mirror and side mirrors were projected onto the screen. The simulator software rendered virtual road environments simulating real world road environments and traffic situations. Data of all relevant parameters were recorded at a rate of 10 Hz. Data was processed and stored in binary data files which were exported to SPSS 24.0 for further analysis.

#### Driving Tasks in the Simulator

Participants performed three types of rides to assess various aspects of driving performance; a lane tracking ride, an intersection ride, and a merging ride. Participants were instructed to drive as they would normally do. The average duration of the lane tracking ride was 5 min, of the intersection ride 7 min, and of the merging ride 3 min on average. Shifting was automatically controlled in all rides. Each ride was performed twice, with the first ride to get the participants familiarized with the simulator and traffic situations. Data from the second rides were used for the analysis.

The lane tracking practice ride was in a rural area on a road with alternating left-right curves of 40 degrees and 500 m radius and a continuous stream of traffic from the opposing direction but no traffic on the lane of the simulator driver. The speed was regulated by the computer and increased stepwise from 50 km/h up to 100 km/h. The test ride was similar to the practice ride with the difference that participants had to control the speed their selves. First participants had to drive with a comfortable speed after which they were asked to drive as if they were in a hurry.

During the intersection rides subjects were confronted with six intersections with different regulations of right-of-way. Participants were always driving straight ahead on the intersections. Speed limits on traffic signs varied between 60 and 80 km/h and participants were asked to apply general traffic rules.

The first and sixth intersections were unmarked with traffic coming from the right and left. In the Netherlands, cars drive on the right side of the road and traffic coming from the right has right of way. The second intersection was also unmarked with traffic only coming from the right so the participant had to give way. The third intersection was unmarked with traffic coming from the left so the participants had right of way. The fourth intersection was marked with a traffic light and participants could continue driving when it turned green. The fifth intersection was marked with a give way sign with traffic coming from the right and left. After the fifth intersection an unexpected event occurred. A parked car pulled out of the parking lot onto the drive way so the participants had to break to avoid a collision. For the analyses intersections 1, 2, 5, and 6 were selected in which subjects had to determine when it was safe to cross (gap distance) by observing the environment in various directions requiring (a combination of) head movements, trunk movements, and eye movements.

The last ride was a merging ride. First subjects needed to merge on the right lane of a highway. Subsequently they had to overtake a car where after they had to return to the right lane. Finally they had to leave the highway and park the car at the side of the road.

Driving in a simulator can provoke motion sickness. Participants were instructed to indicate if they were feeling sick during the driving tasks. If symptoms such as dizziness or nausea were reported or observed, participants were advised to take a break and if symptoms did not reduce to abort the driving simulation.

### Outcome Measures

#### Patient-Related Outcomes

The Tsui scale was used to determine the severity of dystonia (14). The Tsui scale measures different aspects of abnormal posture and movement in CD patients. It has a max score of 25 point where higher scores indicate higher levels of severity.

Perceived muscle fatigue in the neck area and driving effort was rated just after the start and just before the end of the simulated drive in order to determine the effects of drive duration on driving performance, using the Borg CR-10 scale (15). The Borg CR-10 scale is a reliable and validated scale 11 point rating scale, ranging from 0 to 10 on which perceived fatigue and driving effort during the performance of a task can be monitored. For perceived fatigue a score of 0 means that a patient has no muscle fatigue or discomfort in the neck area at all and a score of 10 means there is very much muscle fatigue or discomfort in the neck area. In driving effort a score of 0 means that driving does not require any effort while a score of 10 means that it requires very much effort.

#### Fitness To Drive Screening Questionnaire

Self-perceived fitness to drive was measured with the Fitness To Drive Screening questionnaire (FTDS). The FTDS questionnaire (formerly known as the Safe Driving Behavior Measure) is a self-report questionnaire related to driving fitness, driving behavior and driving safety with a good reliability (ICC.253, *p* < 0.001) (11, 16). The FTDS has three sections: Section A, Demographics or general information about the driver; Section B, Driving history profile; and Section C, Ratings of driving difficulty pertaining to 54 driving skills. Difficulty of the driving task was rated with a 5-point Likert scale (ranging from 1 = extremely difficult to 5 = not difficult at all). A mean score was calculated for each individual by summing up all scores divided by the number of items endorsed.

#### Simulator Outcomes

##### Lane tracking ride

The main measure to assess driving performance in the lane tracking ride was the standard deviation of lateral position (SDLP). The SDLP is the primary outcome for vehicle control and measures the weaving of a car during driving (17). SDLP is considered as the golden standard of driving performance in driving simulators with high test–retest reliability (17).

The SDLP was assessed twice, once for the speed of choice section (SDLP choice) and once for the section participants were asked to drive in a hurry (SDLP hurry). Additional outcomes were average speed, the number of collisions, and percentage of line crossings on either side of the road during each ride.

##### Intersections ride

The main outcome to measures driving performance in the intersection ride were gap distance left and right. Gap distance left and right is defined as the shortest distance from an approaching car coming from the left or right measured in meters. The shorter the distance between the approaching car and the subject, the more risk he or she took to cross the intersection. This also applied for traffic coming from the left which has to give right of way in the Netherlands on unmarked intersections. Traffic coming from the left was programmed to stop to let the subject pass. However, it was also programmed to continue to drive if a subject waited too long.

Additional outcomes on the intersections ride were the lowest speed when approaching the intersection, average deviation from the speed limit and the number of collisions.

##### Merging

In the merging ride the main parameter to measure driving performance was deceleration of the rear car. Deceleration of the rear car was defined by how fast the car behind the participant had to break once a subject merged on the adjacent lane. Deceleration of the rear car depended on distance between the merging and rear car, absolute speed of the rear car and differential speed between the merging and rear car. A larger deceleration of the rear car can be interpreted as a larger risk being taken by the merging car.

Additional outcomes on the merging ride were the speed while merging and rear time headway. Rear time headway was the distance measured in seconds between the subject and car behind the subject once merged on to the adjacent lane. The smaller the time to headway the more risk a participant took to merge.

### Statistical Analysis

First, descriptive statistics (frequencies, means and standard deviations) for patient characteristics were calculated.

Based on the small sample size and unevenly distributed data a Mann–Whitney U was performed to determine differences on simulator outcomes and self-perceived fitness to drive between the patient and control group. To determine the changes in fatigue of the subjects within each group during the driving tasks, a Wilcoxon Signed rank test was used. *P* < 0.05 were considered statistically significant.

## Results

### Participants

A total of 11 patients and 14 controls were included. However, one patient and four controls had to stop the simulator drives due to motion sickness and were excluded from further analysis.

Mean age of the remaining 10 CD patients (5 females, 5 males) had a mean age of 56.8 years (SD 7.7, range 47–69) and a mean disease duration of 15.1 years (SD 11.7, range 1–36 years). Patients showed an average score of 9.7 (SD, 3.1, range 5–14) for the severity of dystonia as measured with the Tsui scale All patients showed a combination of deviating head postures. Eight patients had a rotational component as main direction of the deviating posture (4 patients rotated the left and 4 to the right). Two patients showed a laterocollis as main component of their deviating posture (1 patient had a laterocollis to the left and 1 to the right). The control group existed of 10 persons (6 females, 4 males) with a mean age of 57.3 years (SD 7.4, range 48–67) which were matched for age and gender. Characteristics for each group are described in Table 1.

**Table 1:** Participant characteristics.

	**Patients (*n* = 10)**	**Controls (*n* = 10)**	**Between group difference (*P*-value)**
Age mean (SD, range)	56.8 (7.7, 47–69)	57.3 (7.4, 48–67)	0.747
Gender M/F	5/5	4/6	1.000
Severity of dystonia (Tsui Scale) Mean (SD, range)	9.7 (3.1, 5–14)	NA	NA
Disease duration in years Mean (SD, range)	15.1 (11.7, 1–36)	NA	NA
Driving experience in years Mean (SD, range)	37.5 (9.3, 25–51)	37.4 (8.9, 25–49)	0.990
Driving experience in km/year Range (number of participants)	<1,000 (2) 1,000–5,000 (4) 5,000–10,000 (3) 10,000–50,000 (1) >50,000 (0)	<1,000 (0) 1,000–5,000 (3) 5,000–10,000 (3) 10,000–50,000 (2) >50,000 (1)	0.488
Car accidents in last year	0^*^	0^*^	1.000
Traffic fines in last year Total (range)	1 (0–1)	10 (0–3)^*^	0.028^**^

*NA, Not Applicable*.

* *For 9 out of 10 cases because one did not report the information*.

** *Significant at the < 0.05 level*.

#### Simulator Rides

On the speed of choice section of the lane tracking ride, no significant differences were found on the SDLP. Patients showed a median SDLP of 27.60 cm (range 15.80–35.70) compared to 25.90 cm (range 21.30–43.00) in controls (*p* = 0.853). None of the other outcomes, except the percentage of line crossings on the right side of the road, showed significant differences either. Healthy controls had a significant higher percentage of line crossings than patients. Patients had a median of 0.00 (range 0.00–9.20) percent of the time that they crossed the line compared to a median of 2.30 (range 0.00–37.00) percent of the time in controls (*p* = 0.043). There were no line crossing at the left side of the road.

On the speed in hurry section of the lane tracking ride, none of the outcomes showed significant differences. Patients showed a median SDLP of 28.40 cm (range 20.30–39.70) vs. 31.50 cm (range 21.10–37.80) for healthy controls (*p* = 0.684). An overview of median scores and ranges for all the outcomes and the between group differences are displayed in Table 2.

**Table 2:** Median scores, ranges, and between group difference for driving performance and driving safety.

**Outcome**	**Patients (*N* = 10)i median (range)**	**Control (*N* = 10) median (range)**	**Between group difference (*p*-value)**
**Lane tracking ride**			
*Speed of choice*			
SDLP in cm	27.60 (15.80–35.70)	25.90 (21.30–43.00)	0.853
% line crossings			
Left	(0.00–0.30)	(0.00–0.00)	0.739
Right	(0.00–9.20)	2.30 (00–37.00)	0.043^*^
Nr. of collisions	0	0	NA
Average speed in km/h	79.45 (55.60–86.60)	73.20 (58.00–82.10)	0.280
*Speed in hurry*			
SDLP in cm	28.40 (20.30–39.70)	31.50 (21.10–37.80)	0.684
% line crossings			
Left	(0.00–0.60)	(0.00–0.00)	0.739
Right	(0.00–10.20)	0.70 (0.00–16.20)	0.280
Nr. of collisions	0	0	NA
Average speed in km/h	91.05 (62.90–101.30)	88.60 (74.90–99.70)	0.481
**Intersections ride**			
Gap left int 1 in m	2.78 (1.00–178.44)	1.29 (1.00–164.00)	1.000
Gap left int 2 in m	NA	NA	NA
Gap left int 5 in m^a^	NA	NA	NA
Gap left int 6 in m	1.00 (0.99–8.00)	1.00 (1.00–153.65)	0.741
Gap right int 1 in m	90.64 (3.15–107.23)	72.84 (5.07–123.85)	0.226
Gap right int 2 in m^b^	191.66 (16.29–291.78)	93.92 (7.91–218.66)	0.602
Gap right int 5 in m^c^	NA	NA	NA
Gap right int 6 in m	101.92(2.40–110.67)	98.01 (16.97–138.11)	0.650
Minimum speed int 1 in km/h	(0.00–7.57)	6.00 (0.00–25.49)	0.084
Minimum speed int 2 in km/h	5.17 (1.00–7.79)	4.84 (0.76–7.48)	0.806
Minimum speed int 5 in km/h	(0.00–0.83)	(0.00–0.00)	0.168
Minimum speed int 6 in km/h	2.73 (0.00–6.78)	6.90 (0.00–8.17)	0.070
Deviation from speed limit 60 km/h	1.02 (−5.01–12.81)	2.39 (−5.21–17.26)	0.791
Deviation from speed limit 80 km/h	−6.99 (−31.23–8.21)	−8.75 (−20.83–0.92)	0.734
Nr. of collisions	0	0	NA
**Merging ride on highway**			
*Merging*			
Deceleration rear car merging in km/h	(0.00–0.10)	0.00(−0.20–0.00)	0.169
Speed while merging in km/h	92.50 (88.00–100.70)	97.50 (85.00–111.20)	0.566
Rear time to headway merging in s	1.40 (0.20–2.40)	1.50 (0.20–2.20)	0.965
*Overtaking*			
Deceleration rear car overtaking in km/h	−4.30 (−6.70 to −0.80)	−5.30(−8.00 to −4.00)	0.216
Speed while overtaking in km/h	98.60 (91.50–103.00)	95.60 (91.60–99.70)	0.566
Rear time to headway overtaking in s	0.70 (0.40–1.00)	0.80 (0.20–0.90)	0.559
Minimum time headway overall in s	0.20 (0.13–0.33)	0.28 (0.10–0.42)	0.185

*SDLP, Standard Deviation of the Lateral Position on the road; NA, Not Applicable*.

* *Significant at the P < 0.05 level*.

a *Eight patients and eight controls waited until all oncoming traffic passed before crossing the intersection so gap distance and statistical difference could not be calculated*.

b *Medians, ranges and significance are based on data from 5 patients and 5 controls. Remaining participants waited until all oncoming traffic passed before crossing the intersection so gap distance could not be calculated*.

c *Nine patients and nine controls waited until all oncoming traffic passed before crossing the intersection so gap distance and statistical difference could not be calculated*.

On the intersections ride no significant differences were found on any of the outcomes. On intersection 1 median gap distance with traffic from the left was 2.78 meters (range 1.00–174.44) for patients vs. 1.29 meters (range 1.00–164.00) for controls (*p* = 1.000). Median gap distance with traffic from the right was 90.64 meters (range 3.15–107.23) for patients and 72.84 meters (range 5.07–123.85) for controls (*p* = 0.226). On intersection 2 traffic was only coming from the right. Patients showed a median distance of 191.66 meters (range 16.29–291.78) and controls 93.92 meters (range 7.19–218.66) (*p* = 0.602). On intersection 5 traffic was coming from the left and right where subjects had to give right of way. The majority of the subjects waited until all traffic had passed before crossing the intersection. Eight patients and eight controls waited until all the traffic from the left had passed before crossing the intersection. Nine patients and nine controls waited until all the traffic from the right had passed. Gap distance and statistical differences between the groups could not be calculated for these subjects because there was no gap distance since all the approaching cars had already passed. For these participants gap distance was recorded as a missing value. On intersection 6 median gap distance with traffic from the left was 1.00 meters (range.99–8.00) for patients vs. 1.00 meters (range 1.00–153.65) for controls (*p* = 0.741). Median gap distance with traffic from the right was 101.92 meters (range 2.40–110.67) for patients and 98.01 meters (range 16.97–138.11) for controls (*p* = 0.650).

It was noticeable however that both patients and controls drove with speeds well under the speed limit on the sections where a speed of 80 km/h was allowed (patients median speed 6.99 km/h under the speed limit (range 31.23 km/h under speed limit to 8.21 km/h above speed limit) and controls median speed 8.75 km/h under the speed limit (range 20.83 km/h under speed limit to 0.92 km/h above speed limit), *p* = 0.734).

On the merging ride no significant differences were found on the primary outcome deceleration of the rear car, nor on any of the other additional outcomes. Median deceleration of the rear cars was 0.00 for both patients and controls during merging on the highway (range 0.00–0.10 for patients vs. range −0.20–0.00 for controls, *p* = 0.169). During overtaking rear cars had to decelerate with a median of −4.30 km/h for patients (range −6.70 to −0.80) and for controls with a median of −5.30 km/h (range −8.00 to −4.00, *p* = 0.216).

### Self-Perceived Fatigue and Driving Effort

For self-perceived fatigue, patients showed a median fatigue score of 1.5 at the start of the lane tracking (range = 0.00–6.00) and also 1.5 after finishing (range = 0.00–7.00). For controls a median score of 0.00 was found at the start (range = 0.00–0.00) and a median score of 0.00 after finishing the ride (range = 0.00–5.00). Differences were significantly higher for patients at the start (*p* = 0.005) and finish (*p* = 0.033) of the lane tracking ride, indicating higher levels of fatigue. Although none of the other rides showed significant differences between the groups on self-perceived fatigue, the start of the intersections ride showed a near significant difference (*p* = 0.051) indicating increasing fatigue levels for control subjects during the course of the simulator rides. Neither the patient nor control group showed significant differences in change scores of fatigue before and after each driving task.

A between comparison of the differences between self-perceived fatigue before and after each driving task also did not reveal significant differences on any of the simulator drives.

On the Borg scale for driving effort no significant differences for between the group comparisons, nor for within group comparisons were found for any of the simulator rides. An overview of median scores, ranges, between group differences and within group differences for self-perceived fatigue and driving effort are displayed Table 3.

**Table 3:** Median scores and median change scores of the Borg perceived fatigue, Borg driving effort, and FTDS.

**Outcome**	**Patient median (range)**	**Control median (range)**	**Between group difference (*p*-value)**	**Within group difference (*p*-value)**
*Lane tracking ride*				
Borg perceived fatigue				
• Start of the ride(range)	1.5 (0–6)	0 (0–0)	0.005^*^	Patients: 0.593
• Finish (range)	1.5 (0–7)	0 (0–5)	0.033^*^	Controls: 0.317
• Change score (range)	0 (−3–6)	0 (0–5)	0.957	
Borg driving effort				
• Just after start (range)	1 (0–6)	2.5 (0–7)	0.377	Patients: 0.892
• Just before finish (range)	1 (0–7)	2.5 (0–7)	0.419	Controls: 1.000
• Change score (range)	0 (−3–6)	0 (0–0)	0.619	
*Intersections ride*				
Borg perceived fatigue				
• Start of the ride (range)	1 (0–5)	0 (0–6)	0.051	Patients:0.257
• Finish (range)	2 (0–6)	0 (0–6)	0.095	Controls:0.317
• Change score (range)	0 (−1–4)	0 (0–2)	0.619	
Borg driving effort				
• Just after start (range)	2.5 (0–7)	4 (0–8)	0.515	Patients:0.317
• Just before finish (range)	2.5 (0–8)	4 (0–8)	0.492	Controls: 1.000
• Change score (range)	0 (−1–1)	0 (0–0)	0.278	
*Merging ride on Highway*				
Borg perceived fatigue				
• Start of the ride (range)	1 (0–4)	0 (0–5)	0.400	Patients:0.655
• Finish (range)	1 (0–5)	0 (0–5)	0.220	Controls:0.317
• Change score (range)	0 (−1–4)	0 (−3–0)	0.563	
Borg driving effort				
• Just after start (range)	1 (0–5)	2 (0–8)	0.332	Patients: 1.000
• Just before finish (range)	1 (0–5)	2 (0–8)	0.332	Controls: 1.000
• Change score (range)	0–(0–0)	0 (0–0)	1.000	
FTDS (range)	0.33 (0.01–0.96)	0.21 (0.00–0.74)	0.178	

*FTDS, Fitness To Drive Screening*.

* *Significant at the P < 0.05 level*.

#### Self-Perceived Fitness to Drive

Self-perceived fitness to drive showed a median of individual mean FTDS scores of 0.33 (range 0.01–0.96) for patients and 0.21 for controls (range 0.00–0.74). Group comparison of FTDS scores revealed no significant difference (*p* = 0.178). An overview of median scores, ranges and the between group differences for the FTDS are displayed in Table 3.

## Discussion

In this explorative pilot study, patients with CD did not show significant differences in driving performance as measured with the SDLP on the lane tracking ride, gap distance on the intersections ride and deceleration of the rear car on the merging ride compared to controls. On intersection 5, where the participants had to give way, all but two patients and two controls waited until all approaching traffic had passed before crossing the intersection. Therefore, no gap distance and statistical differences between the groups could be calculated. However, this finding indicates that both patients and controls took no risk with crossing the intersection.

On the additional outcomes, the percentage of line crossings on the right side of the road was significantly higher in healthy controls in the first lane tracking ride, but no differences in line crossings were found between groups in any of the following rides. Self-perceived fatigue was significantly higher for patients compared to controls at the start and finish of the lane tracking ride, but this group difference was not significant in the other driving tasks. No differences between the groups were found in self-perceived fitness to drive.

It was remarkable that no significant differences were found in driving performance in the simulator between the groups, since many patients indicate having trouble with different practical aspects of driving. Patients reported to avoid certain traffic situations like rush hours, bad weather and driving long distances. Patients also indicated that they use tricks while driving in their car such as supporting their head while driving or turning their entire torso to oversee oncoming traffic before crossing an intersection. It was hypothesized that patients would compensate for their physical limitations by driving more carefully and slowly. Studies on compensatory driving behavior indicate that elderly and neurological patients tactically compensate for their limitations by driving slower, increasing the distance to the car in front, waiting longer at intersections, and selecting larger time gaps between the passing cars for merging (13, 18–20). This hypothesis is supported by the patient characteristics were healthy controls had significant more traffic fines mainly for violating the speed limit. The adaptations described in the studies on compensatory driving behavior in elderly and neurological patients were however not observed in the present experiment. This could possibly be explained by overcautious driving behavior in both groups. For example; nine out of ten subjects in each group waited until all traffic coming from the right had passed and drove below the maximum speed while approaching intersection 5 on the intersections ride. It could also be explained by the fact that subjects drove in a research setting where they were watched so they tended to apply the traffic rules much better and gave right of way to anyone coming from the right side. A more general assessment of risk behavior as a personality trait at baseline could have been valuable to exclude that differences in risk behavior may have influenced the results.

Another possible explanation for not finding significant differences on these outcomes is that measurements were performed 4–8 weeks after BoNT injections. With an average working period of 12 weeks and a peak effect between 2 and 4 weeks after injections, we expected an average effect of the BoNT injections. BoNT injections are the treatment of choice in CD, and most patients respond well to treatment which decreases dystonic activity (6). Therefore patients did not have to compensate for the dystonic movements as much and might not have experienced sufficient functional limitations to apply compensatory behavior.

Another possible explanation for not finding significant differences is that CD patients have good coping strategies. CD patients may compensate for impaired control of head movements by moving their eyes or torso instead of their heads while looking for oncoming traffic. Future studies should therefor integrate methods to measure eye movements, gaze behavior, and head movements with the simulator rides to test this hypothesis. Furthermore, the simulator rides were relatively short. This study did not investigate driving performance with prolonged driving, which patients with CD report as problematic. Therefore, the simulator rides were possibly too short to provoke any differences between the groups.

We did find significant differences between groups in fatigue at the start and end of the lane tracking ride. These findings are in line with a study by Smit et al. (3) that showed significant higher levels of fatigue in CD patients measured with the Fatigue Severity Scale compared to health controls. Fatigue, however, did not increase significantly more during the simulator drives in CD patients compared to healthy controls.

The effects of BoNT therapy could explain why there was no significant increase in fatigue during the driving tasks. Due to the decreased dystonic activity in the affected muscles, patients might not have had to compensate for the dystonic movements as much and did not experience a significant increase in fatigue during the driving tasks.

Because this study was an explorative pilot study, there were some limitations.

First of all, the sample size was small so no firm conclusions can be made based on the findings in this study. Secondly, the simulator rides were relatively short and no measures for head or eye movement and gaze direction were incorporated meaning that compensations on this level could not be recorded.

In conclusion, this study compared driving performance between patients with CD and healthy controls on a lane tracking ride, an intersections ride and a merging ride on a highway with a driving simulator. No indications were found that driving performance was significantly different in CD patients compared to healthy controls. However, future studies with larger groups and longer simulator scenarios in which head and eye trackers are applied should be performed to investigate whether these findings can be replicated and to what extent patients compensate for impaired control of head movement. Furthermore, adding groups of patients which are examined during different time points in the BoNT cyclus would be worthwhile to investigate the effect of CD on driving performance in patients with different effects of BoNT.

## Data Availability Statement

The datasets for this article are only available on request. Requests to access the datasets should be directed to Prof. Dr. M.A.J. de Koning-Tijssen, email: m.a.j.de.koning-tijssenmcg.nl.

## Ethics Statement

The studies involving human participants were reviewed and approved by METc UMCG. The patients/participants provided their written informed consent to participate in this study.

## Author Contributions

JD and BV contributed to the study design, data collection, data analysis, data interpretation, figures, and writing of the manuscript. RH contributed to the study design, data collection, data interpretation, and writing of the manuscript. SC contributed to the study design, data analysis, data interpretation, and writing of the manuscript. MT contributed to the study design, data collection, data analysis, data interpretation, and writing of the manuscript.

### Conflict of Interest

The authors declare that the research was conducted in the absence of any commercial or financial relationships that could be construed as a potential conflict of interest. The reviewer, MH, declared a past co-authorship with one of the authors, MT, to the handling editor.
